# Salvage Cryoballoon Ablation After Non-Curative Endoscopic Submucosal Dissection: The First Case Report on T1bN0M0 Esophageal Adenocarcinoma

**DOI:** 10.3390/reports8040265

**Published:** 2025-12-12

**Authors:** Marianna Spinou, Eleni Nakou, Petros Zormpas, Antonis Pikoulas, George Tribonias

**Affiliations:** Department of Gastroenterology, Red Cross Hospital, 11526 Athens, Greece; marspin96@gmail.com (S.M.); elen_nako@hotmail.com (N.E.); pmedcod@gmail.com (Z.P.); antonispik@gmail.com (P.A.)

**Keywords:** esophageal adenocarcinoma, cryoballoon ablation, case report, endoscopic submucosal dissection, Barrett’s esophagus

## Abstract

**Background and Clinical Significance**: Cryotherapy, particularly with the CryoBalloon Focal Ablation System (CbFAS), has emerged as a minimally invasive modality delivering targeted ablation through liquid nitrous oxide. While its role in treating Barrett’s esophagus and dysplasia is well established, its application in early esophageal adenocarcinoma (EAC) salvage treatment remains limited. **Case Presentation**: We report the case of an 84-year-old male with Barrett’s esophagus and multiple comorbidities who underwent endoscopic submucosal dissection (ESD) for a 3 cm esophageal adenocarcinoma (pT1bN0M0). Histology revealed deep submucosal invasion, perivascular infiltration, and positive margins, rendering the resection non-curative. Given surgical ineligibility, the patient underwent cryoballoon ablation six months later for recurrent intramucosal carcinoma proximal to the ESD scar. At three months, surveillance endoscopy showed residual Barrett’s esophagus with low-grade dysplasia. **Conclusions**: This case highlights the feasibility and safety of cryoballoon ablation as salvage therapy after non-curative ESD in inoperable EAC. To our knowledge, this represents the first report of salvage CbFAS in T1bN0M0 EAC, underscoring the need for further studies to define its role in the multimodal management of EAC.

## 1. Introduction and Clinical Significance

Esophageal cancer ranks as the sixth leading cause of cancer-related mortality worldwide, with a 5-year survival rate of less than 25% [1]. Early-stage esophageal adenocarcinoma (EAC), including intramucosal cancers (T1a) and low-risk submucosal tumors (T1b), can be effectively managed with endoscopic resection, reducing both morbidity and mortality [2]. RFA is primarily used for the elimination of low-grade dysplasia without visible lesions in Barrett’s esophagus (BE) as well as following endoscopic resection in order to ablate residual Barrett’s mucosa and prevent recurrence [3]. Non-curative endoscopic resection or high-risk submucosal lesions necessitate adjuvant therapy with salvage esophagectomy and, in some cases, chemoradiotherapy [4,5]. However, salvage surgery after dCRT is associated with substantial perioperative morbidity, including anastomotic leakage, pneumonia with respiratory insufficiency, and sepsis. These complications can prolong hospitalization, increase the need for intensive care, elevate perioperative mortality, and negatively affect health-related quality of life. Managing residual disease in patients unfit for surgery presents a significant clinical challenge. Definitive chemoradiotherapy (CRT) is an appropriate alternative curative option for patients older than 75 years, those with significant comorbidities, or individuals deemed unfit for surgery [6].

Cryotherapy, including cryospray and cryoballoon ablation, utilizes extreme cold to induce tissue necrosis and cell death via intracellular and extracellular freezing, leading to apoptosis and vascular injury [7]. The CryoBalloon Focal Ablation System (CbFAS) delivers cryotherapy through a compliant balloon catheter, allowing precise and circumferential tissue targeting. It has demonstrated efficacy in Barrett’s esophagus (BE) and dysplasia but remains less studied in early esophageal adenocarcinoma (EAC) salvage treatment [8,9]. We present a case of inoperable EAC treated with the CryoBalloon Focal Ablation System (CbFAS) following a non-curative endoscopic submucosal dissection (ESD). This report highlights the potential role of cryoballoon ablation as a salvage therapy in patients who are not candidates for surgical resection.

## 2. Case Presentation

**Figure 1:**
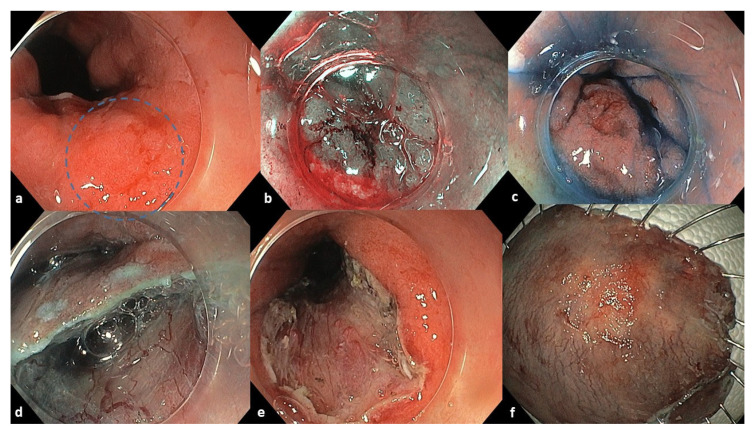
Endoscopic images of the initial lesion and ESD procedure. (**a**) A 3 cm esophageal lesion 0-IIa+c by Paris classification marked inside the blue circle. (**b**) Chromoendoscopy with Narrow Banding Imaging. (**c**) Chromoendoscopy with indigo carmine dye. (**d**) Endoscopic removal of the lesion with single tunneling ESD technique. (**e**) The mucosal defect of the resection. (**f**) Resected tissue specimen.

An 84-year-old male with a history of Barrett’s esophagus (C4M6 by Prague classification), rheumatoid arthritis, and hypertension was referred for the evaluation of a 3 cm polypoid lesion located 38 cm from the incisors. The lesion was classified as 0-IIa+c by Paris classification. Biopsies showed areas of low- and high-grade dysplasia and intramucosal adenocarcinoma. Staging CT scans, a PET scan, and endoscopic ultrasound showed no lymph node or distal metastases (cN0M0). Endoscopic diagnosis in conjunction with EUS classified the lesion as a probable T1. The patient had an ASA score equal to 3 and a Charlson Comorbidity Index (CCI) equal to 7. Endoscopic submucosal dissection (ESD) was performed. The lesion had retracted the muscularis propria, requiring careful dissection. En bloc resection was macroscopically complete (Figure 1). Histopathology revealed a pT1b, moderately to poorly differentiated adenocarcinoma with tubular architecture and superficial ulceration. The tumor invaded 1000 μm into the deep third of the submucosa (SM3, Kikuchi classification) and exhibited perivascular invasion. The lesion reached the deep resection margins while it was extended 1 mm from the proximal horizontal resection margin. Therefore, resection margins were microscopically involved (R1), and high-grade dysplasia was noted proximally. Given the patient’s comorbidities and potential chemotherapy intolerance, the multidisciplinary team recommended adjuvant radiation therapy as the patient was considered medically unfit to tolerate major surgery. The patient was submitted to 45 Gy in total, with 25 fractions of 1.8 Gy.

**Figure 2:**
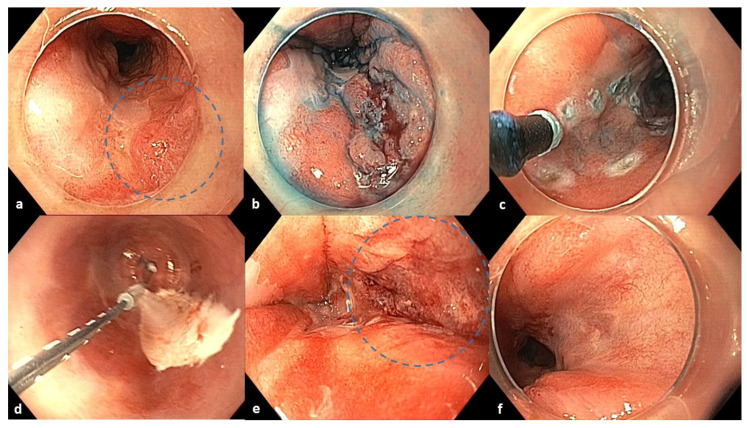
Endoscopic images of the 6-month follow-up after ESD, cryoablation procedure, and 3-month follow-up after cryoablation. (**a**) Six-month follow-up after ESD: homogenous post-ESD scar and a nodular lesion between 3 and 4 o’clock in the blue circle. (**b**) Chromoendoscopy with indigo carmine dye. (**c**) Marking of the lesion with an electrosurgical knife before cryoballoon application. (**d**) Cryoablation with CbFAS system. (**e**) Defect after cryoablation in the blue circle. (**f**) Three-month follow-up after cryoablation: a fibrotic scar at the treated area (4–5 o’clock position).

Six-Month Follow-Up:

Due to patient inability to attend three-month follow up, surveillance endoscopy was performed at six-months showing a clean ESD scar. However, a nodular mucosal lesion between 3 and 4 o’clock proximal to the scar was identified and confirmed as intramucosal carcinoma according to histological analysis confirmed by a second pathologist (Figure 2). Additional staging was carried out with CT scans and endoscopic ultrasound, which revealed no metastatic disease. Therefore, the patient had a Tis-T1N0M0 esophageal adenocarcinoma. Salvage ESD was considered but ultimately ruled out due to the patient’s high-risk profile and technical factors. Specifically, the patient experienced a major cardiovascular event during the initial ESD, rendering him unfit for a repeated prolonged endoscopic procedure. Additionally, the presence of diffuse submucosal fibrosis and non-lifting sign after submucosal injection significantly increased the technical difficulty of the operation. In a redo scenario, these factors would present a prohibitively high risk of complications. The patient, as well as the relatives, were thoroughly informed about the therapeutic options, the technical limitations of a possible ESD, and the advantages and disadvantages of ablation. The cryoballoon ablation system was determined as salvage therapy.

Cryotherapy Treatment:

The CbFAS system (CryoBalloon, Pentax Medical, Redwood City, California, United States) was used under general anesthesia, as shown in the video (online resource). Cryogen (liquid nitrous oxide) was applied for 10 s twice to the neoplastic area and once to the BE segment in a four-quadrant radial and axial pattern (Figure 2). Each application treated an area of ~2 cm^2^. The procedure lasted 10 min. The patient was discharged after 24 h, without complications or dysphagia. Proton pump inhibitors (esomeprazole 40 mg BID), famotidine (40 mg daily), and sucralfate were prescribed for 2 months to optimize mucosal healing.

Three-Month Follow-Up:

White-light endoscopy, NBI, and acetic acid chromoendoscopy revealed residual BE (C0M3) with two 3 cm tongues and a fibrotic scar at the 4–5 o’clock position (Figure 2). Biopsies confirmed low-grade dysplasia. No recurrence or additional neoplastic lesions were observed. A mild distal esophageal stricture—presumably due to extensive cryoablation—was noted but allowed endoscope passage without symptoms (Video S1). The patient was scheduled for radiofrequency ablation (RFA) with the Barrx system for complete BE eradication.

In summary, ESD specimens revealed a pT1bSM3 adenocarcinoma; six-month follow-up, an intrumocal carcinoma; and the three-month post-ablation follow-up, low-grade dysplasia.

## 3. Discussion

This case illustrates the potential of cryoballoon ablation as a salvage treatment following non-curative ESD and radiotherapy in inoperable EAC. Recurrent or residual esophageal cancer after initial therapy with endoscopic resection or chemoradiotherapy (CRT) poses a significant therapeutic challenge. Esophagectomy is considered a definitive treatment for patients with esophageal cancer who are fit for surgery and present residual tumor at the resection specimen margin [10]. CRT after endoscopic resection may serve as a viable approach for frail, elderly patients who cannot tolerate surgery and have non-curatively resected lesions as it prolongs survival rates and minimizes recurrence rates [11,12]. According to Lee et al., close observation could also constitute an option for patients with non-curatively resected superficial esophageal cancer who are medically unable to tolerate further treatment with CRT. For those patients, CRT results in similar 5-year overall survival rates and recurrence rates compared to an exclusively close observation option [13].

Salvage therapeutic options also include repeat endoscopic resection, ablative techniques, and photodynamic therapy (PDT). The feasibility of salvage endoscopic resection, especially that of salvage ESD, is technically challenging and depends on the invasion depth of the lesion, the presence of submucosal fibrosis, and the patient’s surgical candidacy [14,15,16]. Argon plasma coagulation (APC) has also been used for the treatment of patients with recurrent superficial esophageal squamous cell carcinoma after CRT, resulting in survival prolongation [17,18]. PDT has been applicated for recurrent squamous cell esophageal cancer and local failure after CRT/RT, resulting in local disease control [19]. A phase II study revealed an approximately 60% complete response rate and 40% 3-year overall survival in selected patients with no metastatic disease [20]. Nevertheless, most relevant studies consist of case reports or case series for the management of recurrent or residual squamous cell carcinoma, while there are limited data for the palliative treatment of esophageal adenocarcinoma.

Although CbFAS is well established for Barrett’s esophagus, evidence supporting its use in esophageal cancer remains limited. A case reported by Frederiks et al. demonstrated favorable outcomes using CbFAS after incomplete resection with the endoscopic mucosal resection of a high-risk T1b EAC on a patient who was initially diagnosed with a T2N1M0 EAC and treated with chemoradiotherapy [21]. A prospective study carried out on 15 patients with superficial esophageal squamous cell carcinoma on a post-endoscopic resection scar treated with CbFAS revealed a local complete response in all subjects and lesions [22].

Cryotherapy may offer additional benefits such as improved malignancy-associated dysphagia, reduced need for palliative stenting placement, and possible synergy with chemoradiotherapy for subjects with malignant dysphagia from esophageal adenocarcinoma or squamous cell carcinoma [23,24]. It may also enhance immune-mediated tumor response and improve prognosis in solid tumors [25]. In this case, a post-treatment stricture was observed, likely resulting from supratherapeutic dosing. However, cryotherapy preserves extracellular matrix integrity, particularly collagen, reducing stricture risk compared to thermal modalities [26]. A systematic review involving 272 patients and 548 CbFAS treatments reported a 12.5% pooled complication rate (stricture, perforation, bleeding), comparable to RFA and spray cryotherapy [27].

To our knowledge, this is the first report of salvage CbFAS used in a patient with high-risk T1bN0M0 EAC post-nonradical ESD and BE. The patient achieved symptomatic relief and disease control without surgical intervention.

## 4. Conclusions

In conclusion, cryoballoon ablation appears to be a promising salvage option for inoperable esophageal adenocarcinoma after non-curative endoscopic resection. This case supports the need for further prospective studies to evaluate its efficacy, safety, and potential integration into multimodal management strategies for early EAC.

## Data Availability

The original contributions presented in this study are included in the article. Further inquiries can be directed to the corresponding author.

## Supplementary Materials

The following supporting information can be downloaded at https://www.mdpi.com/2571-841X/8/4/265/s1: Video S1. This video includes all the endoscopic interventions and follow-up endoscopic images in detail.

## Abbreviations

The following abbreviations are used in this manuscript:

EAC	Esophageal Adenocarcinoma;
BE	Barrett’s Esophagus;
CbFAS	CryoBalloon Focal Ablation System;
ESD	Endoscopic Submucosal Dissection;
RFA	Radiofrequency Ablation;
CRT	Chemoradiotherapy;
RT	Radiotherapy;
PDT	Photodynamic Therapy;
APC	Argon Plasma Coagulation.

## References

[1] Then E.O., Lopez M., Saleem S., Gayam V., Sunkara T., Culliford A., Gaduputi V. (2020). Esophageal Cancer: An Updated Surveillance Epidemiology and End Results Database Analysis. World J. Oncol..

[2] Schembre D.B., Huang J.L., Lin O.S., Cantone N., Low D.E. (2008). Treatment of Barrett’s esophagus with early neoplasia: A comparison of endoscopic therapy and esophagectomy. Gastrointest. Endosc..

[3] Pech O., Alqahtani S.A. (2020). Update on endoscopic treatment of Barrett’s oesophagus and Barrett’s oesophagus-related neoplasia. Ther. Adv. Gastrointest. Endosc..

[4] Pimentel-Nunes P., Libanio D., Bastiaansen B.A.J., Bhandari P., Bisschops R., Bourke M.J., Esposito G., Lemmers A., Maselli R., Messmann H. (2022). Endoscopic submucosal dissection for superficial gastrointestinal lesions: European Society of Gastrointestinal Endoscopy (ESGE) Guideline—Update 2022. Endoscopy.

[5] Wang A.Y., Hwang J.H., Bhatt A., Draganov P.V. (2021). AGA Clinical Practice Update on Surveillance After Pathologically Curative Endoscopic Submucosal Dissection of Early Gastrointestinal Neoplasia in the United States: Commentary. Gastroenterology.

[6] Faiz Z., Dijksterhuis W., Burgerhof J., Muijs C., Mul V., Wijnhoven B., Smit J., Plukker J. (2019). A meta-analysis on salvage surgery as a potentially curative procedure in patients with isolated local recurrent or persistent esophageal cancer after chemoradiotherapy. Eur. J. Surg. Oncol..

[7] Gage A.A., Baust J. (1998). Mechanisms of tissue injury in cryosurgery. Cryobiology.

[8] Visrodia K., Zakko L., Singh S., Leggett C.L., Iyer P.G., Wang K.K. (2018). Cryotherapy for persistent Barrett’s esophagus after radiofrequency ablation: A systematic review and meta-analysis. Gastrointest. Endosc..

[9] Schölvinck D.W., Künzli H.T., Kestens C., Siersema P.D., Vleggaar F.P., Canto M.I., Cosby H., Abrams J.A., Lightdale C.J., Tejeda-Ramirez E. (2015). Treatment of Barrett’s esophagus with a novel focal cryoablation device: A safety and feasibility study. Endoscopy.

[10] Wang W.-P., Ni P.-Z., Yang J.-L., Wu J.-C., Yang Y.-S., Chen L.-Q. (2018). Esophagectomy after endoscopic submucosal dissection for esophageal carcinoma. J. Thorac. Dis..

[11] Kawaguchi G., Sasamoto R., Abe E., Ohta A., Sato H., Tanaka K., Maruyama K., Kaizu M., Ayukawa F., Yamana N. (2015). The effectiveness of endoscopic submucosal dissection followed by chemoradiotherapy for superficial esophageal cancer. Radiat. Oncol..

[12] Hamada K., Ishihara R., Yamasaki Y., Hanaoka N., Yamamoto S., Arao M., Suzuki S., Iwatsubo T., Kato M., Tonai Y. (2017). Efficacy and safety of endoscopic resection followed by chemoradiotherapy for super-ficial esophageal squamous cell carcinoma: A retrospective study. Clin. Transl. Gastroenterol..

[13] Lee J.W., Cho C.J., Kim D.H., Ahn J.Y., Lee J.H., Choi K.D., Song H.J., Park S.R., Lee H.J., Kim Y.H. (2018). Long-Term Survival and Tumor Recurrence in Patients with Superficial Esophageal Cancer after Complete Non-Curative Endoscopic Resection: A Single-Center Case Series. Clin. Endosc..

[14] Hwang C., Youn Y.H., Choi S.-E., Jung Y.H., Park H.Y., Park J.J., Kim J.H., Park H. (2015). Endoscopic Submucosal Dissection for Recurrent or Residual Superficial Esophageal Cancer after Chemoradiotherapy: Two Cases. Clin. Endosc..

[15] Saito Y., Takisawa H., Suzuki H., Takizawa K., Yokoi C., Nonaka S., Matsuda T., Nakanishi Y., Kato K. (2008). Endoscopic submucosal dissection of recurrent or residual superficial esophageal cancer after chemoradiotherapy. Gastrointest. Endosc..

[16] Yano T., Muto M., Hattori S., Minashi K., Onozawa M., Nihei K., Ishikura S., Ohtsu A., Yoshida S. (2008). Long-term results of salvage endoscopic mucosal resection in patients with local failure after definitive chemoradiotherapy for esophageal squamous cell carcinoma. Endoscopy.

[17] Noordzij I.C., Curvers W.L., Huysentruyt C.J., Nieuwenhuijzen G.A., Creemers G.-J., van der Sangen M.J., Schoon E.J. (2018). Salvage endoscopic resection in patients with esophageal adenocarcinoma after chemoradiotherapy. Endosc. Int. Open.

[18] Matsutani T., Nomura T., Hagiwara N., Matsuda A., Uchida E. (2017). Salvage Endoscopic Argon Plasma Coagulation After Chemoradiotherapy for Inop-erable Esophageal Cancer. Surg. Laparosc. Endosc. Percutan. Tech..

[19] Nishikawa M., Yamamoto Y., Kushida S., Hirabayashi T., Tanaka S., Takegawa N., Mimura T., Tsumura H., Miki I., Tsuda M. (2022). Assessment of photodynamic therapy as a salvage treatment for local failure after chemoradiotherapy or radiotherapy for esophageal cancer in patients aged 80 years or older. DEN Open.

[20] Yano T., Kasai H., Horimatsu T., Yoshimura K., Teramukai S., Morita S., Tada H., Yamamoto Y., Kataoka H., Kakushima N. (2017). A multicenter phase II study of salvage photodynamic therapy using talaporfin sodium (ME2906) and a diode laser (PNL6405EPG) for local failure after chemoradiotherapy or radiotherapy for esophageal cancer. Oncotarget.

[21] Frederiks C.N., van de Water J.M.W., Ebrahimi G., Weusten B.L.A.M. (2022). Cryoballoon ablation as salvage therapy after nonradical resection of a high-risk T1b esophageal adenocarcinoma: A case report. Eur. J. Gastroenterol. Hepatol..

[22] Sunakawa H., Yoda Y., Nonaka S., Suzuki H., Abe S., Ishiguro Y., Ikeno T., Wakabayashi M., Sato A., Nakajo K. (2024). Prospective multicenter trial of the cryoballoon ablation system for superficial esophageal squamous cell carcinoma on post–endoscopic resection scars: A CRYO-SCAR study (EPOC1902). Gastrointest. Endosc..

[23] Kachaamy T., Prakash R., Kundranda M., Batish R., Weber J., Hendrickson S., Yoder L., Do H., Magat T., Nayar R. (2018). Liquid nitrogen spray cryotherapy for dysphagia palliation in patients with inoperable esophageal cancer. Gastrointest. Endosc..

[24] Hanada Y., Leggett C.L., Iyer P.G., Linn B., Mangels-Dick T., Wang K.K. (2022). Spray cryotherapy prevents need for palliative stenting in patients with esophageal cancer-associated dysphagia. Dis. Esophagus.

[25] Shah T., Kushnir V., Mutha P., Majhail M., Patel B., Schutzer M., Mogahanaki D., Smallfield G., Patel M., Zfass A. (2019). Neoadjuvant cryotherapy improves dysphagia and may impact remission rates in advanced esophageal cancer. Endosc. Int. Open.

[26] Evonich R.F., Nori D.M., Haines D.E. (2007). A randomized trial comparing effects of radiofrequency and cryoablation on the structural integrity of esophageal tissue. J. Interv. Card. Electrophysiol..

[27] Westerveld D.R., Nguyen K., Banerjee D., Jacobs C., Kadle N., Draganov P.V., Yang D. (2020). Safety and effectiveness of balloon cryoablation for treatment of Barrett’s associated neoplasia: Systematic review and meta-analysis. Endosc. Int. Open.

